# Castleman's Disease: A Case Report of the Unicentric Type

**DOI:** 10.1155/2012/175272

**Published:** 2012-09-10

**Authors:** J. Gomez-Ramirez, M. Posada, L. Sanchez-Urdazpal, E. Martin-Perez, L. Del Campo, I. Garcia, J. L. Martin, E. Larrañaga

**Affiliations:** ^1^Department of General Surgery, La Princesa University Hospital, 62 Diego de Leon Street, 28006 Madrid, Spain; ^2^Department of Radiology, La Princesa University Hospital, 62 Diego de Leon Street, 28006 Madrid, Spain

## Abstract

Castleman's disease, or angiofollicular lymph node hyperplasia, is a relatively rare disorder characterized by the benign proliferation of lymphoid tissue related to the chronic human herpes virus 8 (HHV-8) infection and the human immunodeficiency virus (HIV). Two clinical entities have been described: a unicentric presentation with the disease confined to a single anatomic lymph node and a multicentric presentation characterized by generalized lymphadenopathy and a more aggressive clinical course. Also, three histopathological subtypes have been described: hyaline-vascular, plasma cell, and a mixed variant. Preoperative diagnosis of hyaline-vascular Castleman's disease is difficult, and the definitive result is based on postoperative pathological findings. The gold standard therapy is the complete surgical excision.

## 1. Castleman's Disease: A Case Report of the Unicentric Type

Castleman's disease (CD) is an uncommon disorder characterized by a benign proliferation of the lymphoid tissue that may be localized or unicentric (UCD) or disseminated or multicentric (MCD). Histologically, CD can be classified as hyaline-vascular type, plasma cell type, or a mixed type [1]. Patients with localized hyaline-vascular type are usually asymptomatic and are diagnosed during routine imaging studies. The definitive diagnosis is based on postoperative pathological findings. The aim here was to describe a case of retroperitoneal unicentric Castleman's disease, its diagnostic tools, and the perioperative management.

## 2. Report of a Case

**Figure 1:**
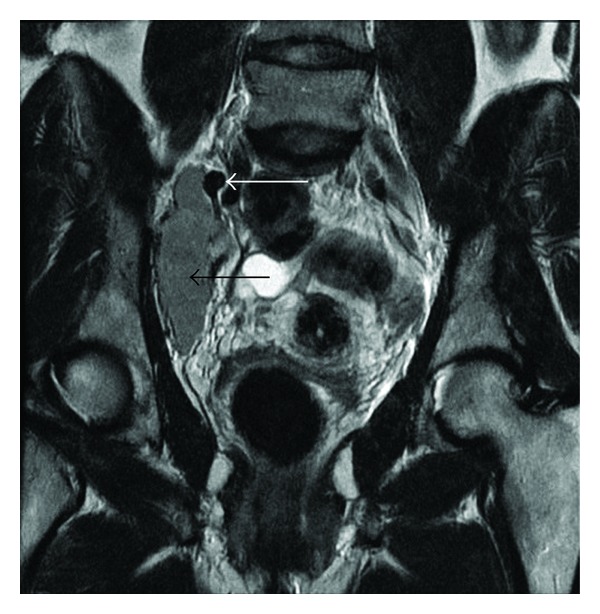
Pelvic magnetic resonance imaging: coronal T2-weighted imaging shows a well-capsulated, hyperintense, solid mass (black arrow), next to the iliac vessels (white arrow).

**Figure 2:**
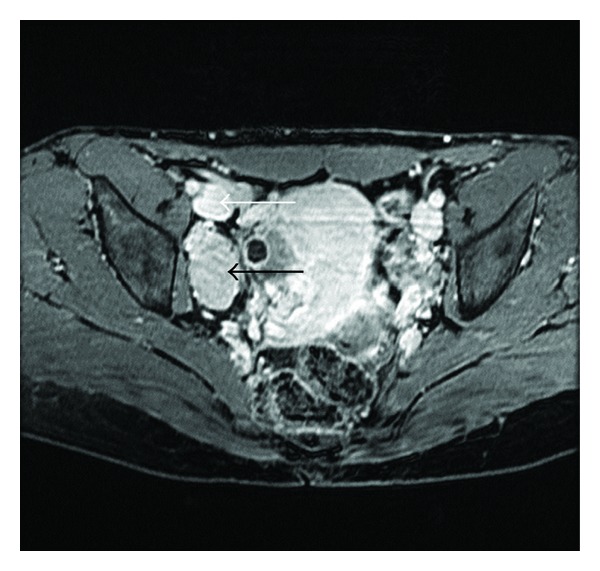
Pelvic magnetic resonance imaging: axial GE-T1 fat sat with gadobenate dimeglumine in venous phase shows intense enhancement (black arrow), similar to the vessels (white arrow).

**Figure 3:**
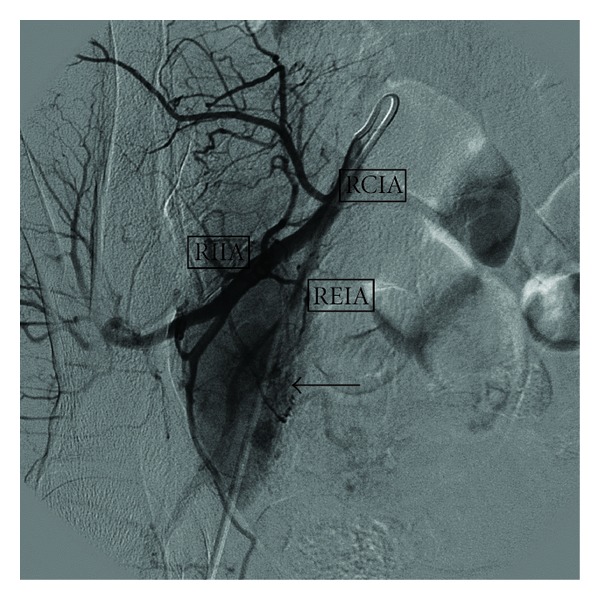
Selective right iliac arteriography prior to embolization, that demonstrates pront enhancement of the lesion (arrow) after contrast infusion. RCIA: right common iliac artery, REIA: right external iliac artery, RIIA: right internal iliac artery.

A 47-year-old woman with no previous medical history was evaluated at our hospital due to an incidental pelvic mass found during a routine gynaecological ultrasound. No associated fever, vomiting, or localized abdominal pain had occurred and the physical examination, blood test, and abdominal X-ray were all normal. The abdominal ultrasound showed a 60 mm hypoechoic retroperitoneal mass located between the right iliac vessels bifurcation.The RM study revealed a 7 × 4 cm homogeneous mass of soft-tissue attenuation, iso-intense to muscle in signal intensity on T1-weighted and hyper-intense on T2-weighted MR imaging with intense and homogeneous enhancement in dynamic contrast sequences (Figures 1 and 2). A subsequent fine needle aspiration (FNAB) showed a lymph node with a reactive hyperplasia cell pattern. Preoperative embolization of the feeding arteries was performed to decrease intraoperative bleeding (Figure 3). At laparotomy, a solid mass was found attached to the right iliac vein bifurcation and a complete resection of the mass was undertaken. Pathologic exam of the lesion revealed a hyaline-vascular type of Castleman's disease. The patient had an uneventful postoperative course. A 3 month follow-up CT did not disclose any other thoracic or intra-abdominal lesions.

## 3. Comment

CD was first described in 1956 by Benjamin Cattleman, who identified a group of patients with solitary hyperplasic mediastinal lymph nodes with small germinal center resembling Hassall's corpuscles of the thymus [2]. These lymph nodes had small, prominent, hyalinized follicles associated with a marked interfollicular vascular proliferation (hyaline vascular variant of CD). This type of disease is now known as Unicentric Castleman's Disease (UCD).

MCD is a systemic disease characterized by fever and night sweats associated with generalized peripheral lymphadenopathy and hepatosplenomegaly, which is frequently related to the plasma cell variant [3]. Although the occurrence rate is unknown, it is increasingly relevant nowadays due to its association with HIV and HHV-8 [4]. MCD has also been associated with other malignancies, in particular Kaposi's sarcoma and lymphomas. Most patients with MCD die from progression of their disease, disseminated infection, or related malignancies.

A variety of treatments have been used for MCD including surgery, radiation, steroids, antiviral agents, specific antibodies, inhibitors of cytokines activity, and chemotherapy [5]. Surgery generally does not have a role in the treatment of MCD, although splenectomy may result in temporary symptomatic improvement.

UCD is the most common type and consists of an isolated benign lymphoproliferative disorder of young adults that is not associated with an HHV-8 infection and usually curable with surgical resection. The vast majority of patients are asymptomatic and their disease is identified incidentally on imaging studies as a soft tissue mass located in the neck or mediastinum and rarely in the retroperitoneum, as the case herein reported. This particular location of UCD caused a troublesome differential diagnosis due to nonspecific clinical signs and radiological features. Preoperative diagnosis of hyaline-vascular Castleman's disease is difficult. The usual appearance of this entity on a CT or MR is that of a nonspecific homogeneous mass on noncontrasted studies, with dense enhancement immediately after the infusion of iodinated or gadolinium material and slow washout with the degree of enhancement approaching that of large vessels [6]. The presence of central areas of fibrosis of this tumour is one of the characteristic features of this disease [7]. FNAB is usually nondiagnostic or indicative of reactive lymphadenopathy. The definitive diagnosis is based on postoperative pathological findings. Once CD is diagnosed, MCD must be ruled out. In addition, UCD may be associated with an increased risk of lymphoma (B-cell non-Hodgkin's lymphoma and Hodgkin lymphoma) [8].

The standard therapy for UCD hyaline-vascular form of CD is surgical excision, and since these are hypervascular lesions, it is frequently associated with profuse bleeding, and preoperative embolization can help to minimize intraoperative blood loss [9]. Surgery is curative when resection is complete, yielding a 5-year survival rate close to 100%, with recurrences being infrequent [10]. In patients whose lesions cannot be completely resected, outcomes remain favourable. Partially resected masses may remain stable and asymptomatic for many years [11]. Radiation therapy may result in complete or partial remission rates of 40 and 10 percent, respectively. Also successful use of rituximab has been reported [12]. Appropriate followup should be tailored to the specific CD variant and symptoms. Patients with unicentric disease without systemic involvement should have an additional radiological assessment six to twelve months after initial therapy, to verify there is not recurrence. Additional testing or therapy should only be pursued in the event of recurrence or the onset of new symptoms.
